# Retrospective study of efficacy and adverse events of immune checkpoint inhibitors in 22 xeroderma pigmentosum patients with metastatic or unresectable cancers

**DOI:** 10.3389/fonc.2023.1282823

**Published:** 2023-10-25

**Authors:** Elvelyn R. Fernandez, Deborah Tamura, Sikandar G. Khan, Sophie Momen, Hiva Fassihi, Robert Sarkany, John J. DiGiovanna, Kenneth H. Kraemer

**Affiliations:** ^1^ DNA Repair Section, Laboratory of Cancer Biology and Genetics, Center for Cancer Research, National Cancer Institute, Bethesda, MD, United States; ^2^ National Xeroderma Pigmentosum Service, St John’s Institute of Dermatology, Guy’s and St Thomas’ NHS Foundation Trust, London, United Kingdom

**Keywords:** xeroderma pigmentation, cancer, immune checkpoint inhibitor (ICI), genodermatosis, UV radiation, melanoma, squamous cell carcinoma, immunotherapy

## Abstract

**Background:**

Xeroderma pigmentosum (XP), a rare disease with defects in DNA repair genes, has >1,000-fold increased risk of ultraviolet-induced skin cancers. Immune checkpoint inhibitors (ICIs) are used for treating cancers with large numbers of mutations but may also promote adverse events (AEs). Deficient DNA repair in XP patients may lead to increased numbers of mutations, leading to enhanced efficacy of cancer response or, alternatively, to increased AE in response to ICI. We sought to compare the efficacy and AE of ICI in XP patients with metastatic or unresectable cancers to that of ICI-treated patients in the general population.

**Methods:**

In this retrospective study, we reviewed medical records of XP patients treated in the United States and in London (UK). We also reviewed published reports of ICI-treated XP patients and patients in the general population.

**Results:**

Metastatic or unresectable cancers in all 22 (100%) XP patients showed regression or remission in response to ICI. The types and frequencies of AE in XP patients were similar to those reported among ICI-treated patients in the general population. However, two XP patients had concurrent additional cancers that did not respond to ICI, two XP patients had cancer recurrence or progression after initial response, and eight XP patients developed new skin cancers during or after ICI treatment.

**Conclusion:**

In this retrospective study with small sample size, XP patients demonstrated positive responses to ICI and the treatment was well tolerated but some patients developed new skin cancers while being treated. ICIs can be considered in treating metastatic or unresectable cancers in XP patients.

## 1 Introduction

Xeroderma pigmentosum (XP) is a rare autosomal recessive disease affecting about one in a million people in the United States (US) and Europe. XP patients sunburn easily and develop freckle- like hyperpigmented macules before age 2 years (1). These patients have mutations in genes involved in repairing ultraviolet (UV)–induced DNA damage. Because of failure to repair DNA damage, XP patients’ cells may harbor large numbers of mutations (2–4). Their sensitivity to UV results in a >1,000-fold increased risk of skin cancers and 34-fold increased risk of internal tumors (5, 6).

Evasion of the immune system is one cause of cancer growth (7). Immune checkpoint inhibitors (ICIs) promote the T-cell antitumor response by blocking immune checkpoints such as the protein receptor cytotoxic T-lymphocyte–associated protein 4 (CTLA-4) on T cells. In 2011, the US Food and Drug Administration (FDA) approved the use of the first ICI drug ipilimumab, a monoclonal antibody that targets CTLA-4 (8). The FDA has approved other ICI drugs targeting T-cell-programmed cell death protein 1 (PD-1) (pembrolizumab, nivolumab, and cemiplimab), programmed cell death ligand 1 (PD-L1) (atezolizumab, avelumab, and durvalumab), and CTLA-4 (tremelimumab) (9) However, ICI may also activate T cells that target non-cancer tissues, thus resulting in immune-related adverse events (irAE) (10). ICIs have been shown to be effective at treating cancers with high tumor mutational burden (defined as ≥10 mutations per megabase) (11–13).

Currently, effective management of XP consists of sun protection, cancer screenings, and treatment of cancers with topical drugs (5-fluorouracil or imiquimod) and surgery (1, 14). As ICIs become more commonly used within the general population, it is important to assess the potential benefits and risks of using ICIs to treat XP patients. Deficient DNA repair in XP patients can lead to increased mutations in XP cancers and may enhance the efficacy of ICI. On the other hand, increased mutations in non-cancer tissues may promote autoimmunity (15), leading to increased off-target adverse events (AEs).

We performed a retrospective study by reviewing medical records of XP patients and analyzing the available literature. We evaluated the efficacy and AE of ICI in XP patients compared to ICI-treated patients in the general population. We also collected information on tumor mutational burden in XP patients.

## 2 Article type—original research

## 3 Patients and methods

XP patients in the US were referred by their local healthcare providers for enrollment in a natural history study at the National Institutes of Health (NIH) (National Cancer Institute protocol 99C0099). Patients were examined at the NIH Clinical Center. Patients subsequently developed cancers that were treated with ICIs by their local doctors. The ICI treatment selection and evaluation were made by their local doctors. After they were treated, we obtained patients’ medical records from the institutions where they received ICI treatment. XP patients in the United Kingdom (UK) were under the care of the National XP Clinic at Guy’s and St Thomas’ Foundation Trust in London (16). Methods of data collection and assessment of response, AE, and irAE (17–20) are described in Appendix S1. The ICI target cancer selection and treatment and the response to ICI treatment was determined by local physicians. Treatment response was based on tumor imaging from positron emission tomography (PET), computed tomography (CT), magnetic resonance imaging (MRI), or clinical photography.

Case reports of ICI-treated XP patients and studies of ICI-treated patients in the general population were found through the search engines PubMed and scite (http://scite.ai). Available information on patients’ medical histories, tumor descriptions, ICI treatment courses, AE, and tumor mutational burden were noted and compared with the NIH and UK cohorts of XP patients (**Supplementary Appendix S1**).

## 4 Results

### 4.1 ICI responses of 22 XP patients from NIH, UK, and literature review

**Figure 1:**
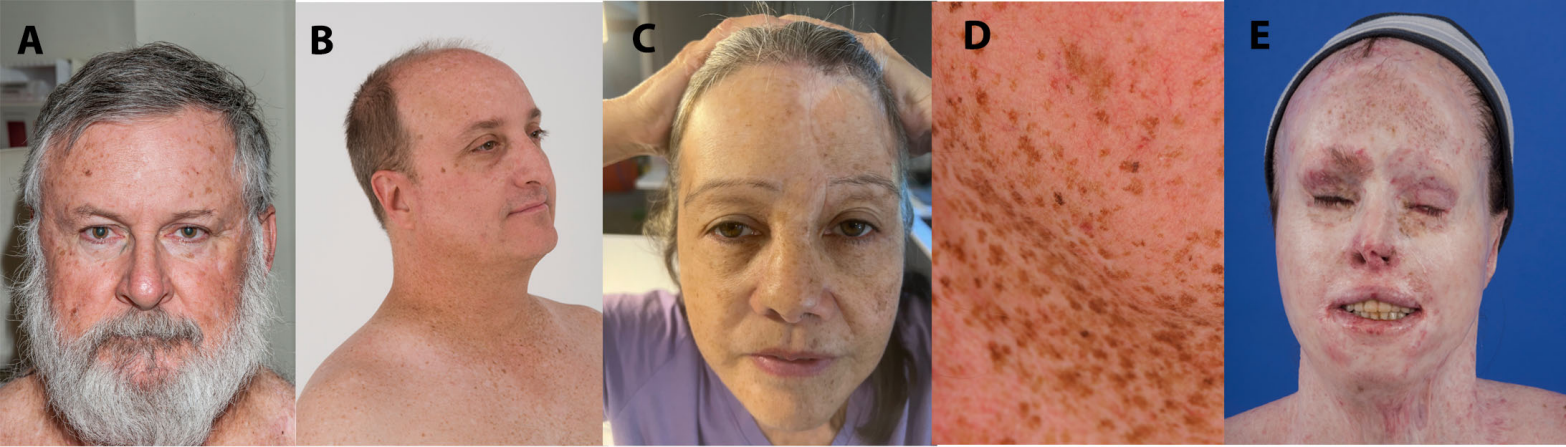
XPC patients demonstrating typical scarring from skin cancer surgeries **(A)** and facial freckling **(B–D)**. **(A)** XP495BE pictured at age 61 years. He was treated with nivolumab for multiple primary melanomas (without metastasis) at age 63 years. **(B)** XP9BE pictured at age 51 years. He was treated with pembrolizumab for metastatic melanoma at age 57 years. **(C)** XP675BE pictured at age 61 years. She was treated with nivolumab for metastatic amelanotic melanoma at age 58 years. **(D)** XP376BE pictured at age 45 years. She was treated with pembrolizumab for metastatic NSCLC at age 59 years. **(E)** XP572BE pictured at age 32 years. She was treated with pembrolizumab then nivolumab for metastatic melanoma at age 35 years.

**Figure 2:**
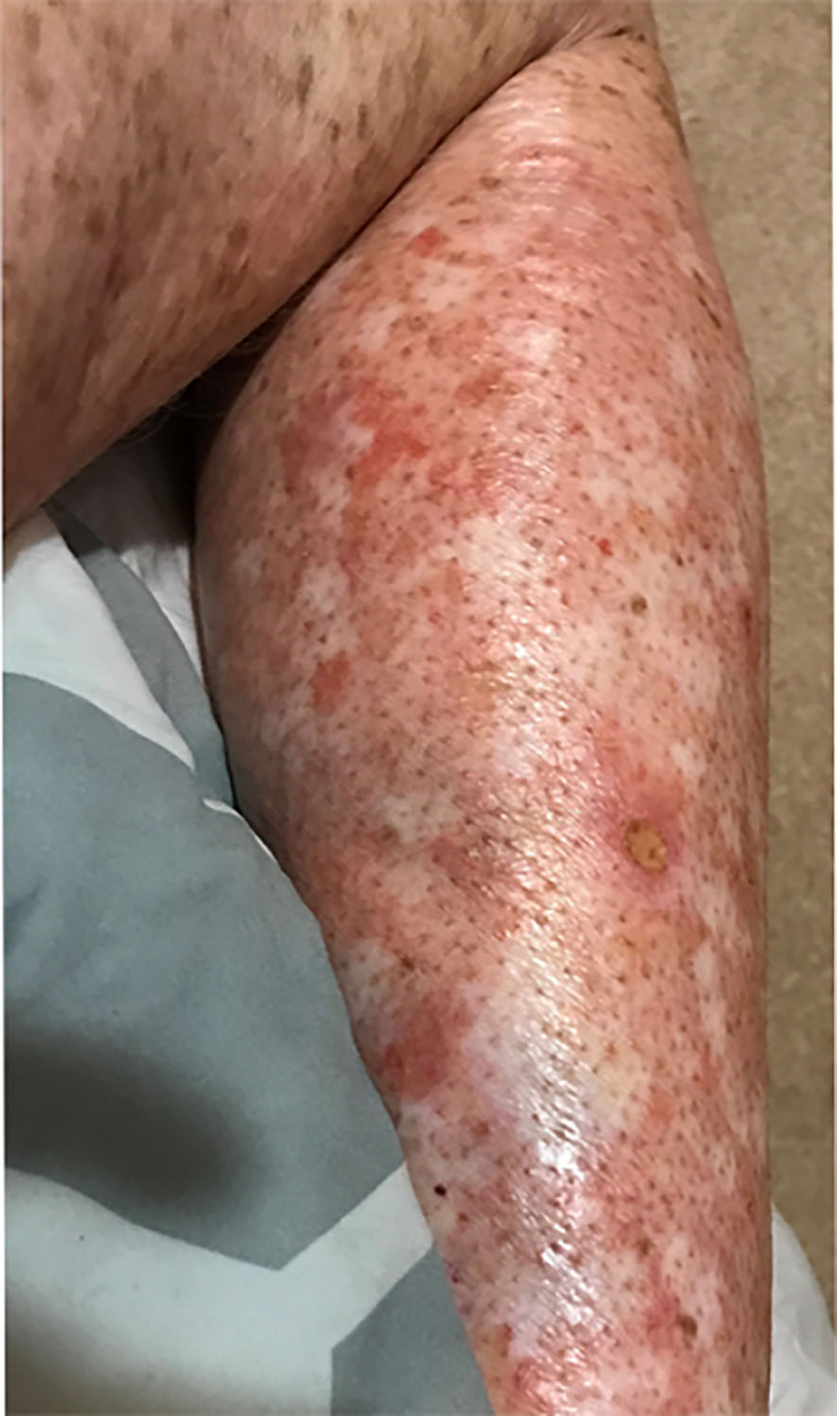
XP572BE CTCAE Grade 3 eczematous dermatitis on right lower leg secondary to immunotherapy.

Within the NIH, UK, and published case reports (21–31), there were 22 XP patients treated with ICI: 14 XP-C patients (*XPC* gene), one XP-D patient (*ERCC2* gene), two XP-E patients (*DDB2* gene), three XP variant patients (*POLH* gene), and two patients with unreported XP mutations (**Table 1**; **Supplementary Tables S1–S3**). Five XP-C patients enrolled at the NIH displayed typical XP characteristics of facial freckling and scarring from skin cancer surgeries (**Figures 1**, **2**). XP patients were treated for melanoma (11 cases), squamous cell carcinoma (SCC) (eight cases), non-small cell lung cancer (one case), angiosarcoma (one case), or sarcomatoid carcinoma (one case on scalp) (**Table 1**). One patient had metastatic melanoma and SCC treated with ICI at different times (21).

**Table 1:** Responses of 22 XP patients from NIH, UK, and literature review to immune checkpoint inhibitors.

XP COHORT	NUMBER OF PATIENTS	TARGET CANCERS	ICI TREATMENT	PATIENTS WITH TARGET CANCER REGRESSION OR REMISSION FROM ICI n/total (%)	PATIENTS WITH NEW CANCERS* DETECTED DURING OR AFTER ICI n/total (%)
NIH	6	Metastatic melanoma, Multiple primary melanomas (without metastasis), Metastatic non-small cell lung cancer	Nivolumab, Pembrolizumab	6/6 (100%)	4/6 (67%)
UK	3	Metastatic melanoma, Metastatic angiosarcoma	Nivolumab, Pembrolizumab	3/3 (100%)	1/3 (33%)
Literature Review	13	Metastatic melanoma, Sarcomatoid carcinoma of the scalp (without metastasis), Metastatic SCC, Ocular and cutaneous SCC (without metastasis), Angiosarcoma (without metastasis; patient also had BCC of the right face)	Nivolumab, Pembrolizumab, Ipilimumab, Cemiplimab	13/13 (100%)	3/13 (23%)
**Total**	**22**			**22/22 (100%)**	**8/22 (36%)**

XP, xeroderma pigmentosum; ICI, immune checkpoint inhibitor; NIH, National Institutes of Health; UK, United Kingdom; National Xeroderma Pigmentosum Clinic

*Patients had new cancers of the following types: melanoma, basal cell carcinoma, and squamous cell carcinoma.

Before ICI treatment, seven patients with metastatic melanoma (two patients), sarcomatoid carcinoma (one patient (23)), metastatic cutaneous SCC (cSCC) (two patients (27, 28))), and unresectable or metastatic angiosarcoma (two patients (22, 32) did not respond to treatment with cytokines, chemotherapy, radiotherapy, targeted antibody therapy (**Supplementary Tables S1–S3**). In contrast, all 22 patients in the study showed regression or remission of the target cancer in response to ICI (treatment duration ranged from 2 to 60 months) (**Table 1**). Patients had been followed and remained in regression or remission for 2 to 119 months after their first cycle of ICI (ongoing ICI treatment), after resuming ICI treatment (ongoing treatment), their last cycle of ICI, or their first PET/CT showing no evidence of metastasis (**Supplementary Tables S1–S3**).

Two XP patients had additional cancers that did not respond to ICI. An XP patient had more than one type of cancer (angiosarcoma and BCC) treated at the same time with ICI. The angiosarcoma completely responded to ICI. However, the BCC did not respond to ICI and was instead treated with sonic hedgehog inhibitor vismodegib and surgery (22). Another XP patient was treated with pembrolizumab for multiple SCC tumors (left lower eyelid, right conjunctiva and cornea, right preauricular masses, and right parotid lymph node). Cutaneous and mucous membrane SCC tumors regressed with ICI treatment. However, the right corneal tumor showed mild progression during ICI treatment and was then treated with topical 5-fluorouracil (31).

Two XP patients had cancer recurrence or progression after a period of responding to ICI. An XP patient showed remission of lymph node metastases from cSCC after two courses of ICI and stopped ICI treatment after 4 months. Eighteen months later, he developed a recurrence of cSCC on the neck and restarted ICI treatment. At the time of publication, the patient showed remission of the recurrent cSCC (28). Another XP patient showed remission of a metastatic melanoma treated with ipilimumab and regression of an unresectable maxillary sinus SCC treated with pembrolizumab. However, 31 months after the first pembrolizumab cycle, the SCC progressed and pembrolizumab was discontinued (21).

Eight patients developed new localized, primary cancers (melanoma, basal cell carcinoma—BCC, SCC) during or after ICI treatment (**Table 1**). One patient developed new superficial lesions on the scalp, tongue, and right auricle after his first course of ICI (28).

We are presenting details of XP9BE who showed progression of his metastatic cancer in response to treatment with cytokines and radiotherapy and showed cancer remission in response to ICI. Case presentations of the other five XP patients in the NIH cohort (33–36) and 1 XP patient in the UK cohort (32, 37, 38) are detailed in **Supplementary Appendices S2, S3**; **Supplementary Figures S2, S3**.

### 4.2 Patient XP9BE

Patient XP9BE (**Figure 1**) was an XP-C patient diagnosed at age 6 years. He had a twin brother who died at age 32 years due to metastatic melanoma and an older sister with XP who died at age 63 years due to ovarian cancer. He had two older siblings living with XP (39, 40). He had surgical treatment of his first melanoma at age 19 years. At age 45 years, he had a stage III melanoma on his left ear with lymph node, scalp, and nasolabial crease metastases treated with surgery and interferon (**Supplementary Figure S2C**). From ages 45–54 years, he had a BCC, one intraoral cheek melanoma (unspecified stage), and two melanomas *in situ*. At age 55 years, he developed a 2-cm melanoma on the right side of his face with a right parotid gland metastasis. Following surgery and adjuvant recombinant granulocyte macrophage colony-stimulating factor (GM-CSF) treatment, he developed recurrent in-transit melanoma on his right head and neck region. Adjuvant radiation therapy (31 rounds) was performed after excision of the head and neck metastases. At age 57 years, new melanoma metastases were discovered in his head and neck, lungs, and mediastinum. He was treated with 34 cycles of pembrolizumab (**Supplementary Table S1**; **Supplementary Figure S2C**). Four months after his first cycle of pembrolizumab, there was no evidence of tumors found on PET scan. This complete response has persisted for 60 months. During ICI treatment, the patient experienced mild AE of subclinical hypothyroidism and fatigue and developed an invasive cSCC on his forehead which was removed through Mohs surgery (**Supplementary Table S1**). After ICI treatment, he had an Eastern Cooperative Oncology Group Performance Status of Grade 0. 140

### 4.3 Frequency of AE in 22 XP patients and general population treated with ICI

Within the NIH, UK, and literature review cohorts, 11 of 22 XP patients (50%) developed any-grade AE (Common Terminology Criteria for Adverse Events—CTCAE Grades 1–5) up to 31 months after beginning ICI treatment (**Table 2**). Two XP patients (10%) developed severe, CTCAE Grades 3–4 AE within 0.5–8 months after beginning ICI (**Table 2**) and discontinued their ICI treatment (XP495BE and XP572BE). No XP patients died with ICI treatment. Among XP patients with AE, four patients (18%) developed irAE that were evidenced by immunological, serological, or histological data. The irAE consisted of CTCAE Grades 3–4 encephalitis (*n* = 1), Grade 2 hypothyroidism (*n* =1), Grade 3 rash and pruritus (*n* = 1), and Grade 1 vitiligo (*n* = 1) arising within 0.5–12 months after beginning ICI treatment (**Table 2**).

**Table 2:** Frequencies of AE in 22 XP patients and general population treated with ICI.

CATEGORY	AE FREQUENCY IN XP [n/total]	AE FREQUENCY IN GENERAL POPULATION [n/total]*
Any-grade AE (CTCAE Grades 1–5)	50% [11/22]	78% [632/811] (19)
CTCAE Grades 3–5 AE	9% [2/22]#	18% [146/811] (19)
Delayed onset AE/irAE (arising > 12 months after beginning ICI treatment)	9% [2/22]	5% [53/999] (41)
Fatigue	23% [5/22]	23% [188/811] (19)
Cutaneous AE/irAE	27% [6/22]	25% [2171/8637] (42)
Pruritus	9% [2/23]	5% [416/8637] (42)
Neurological irAE	5% [1/22]	1% [35/3763](50)
Rheumatic AE/irAE	14% [3/22]	7% [35/524](51)
Endocrinopathies	18% [4/22]	12% [40/339](52)
Liver enzyme elevation	9% [2/22]	4% [17/470](53)

XP, xeroderma pigmentosum; ICI, immune checkpoint inhibitors; AE, adverse events; CTCAE, Common Terminology.

Criteria for Adverse Events; irAE, immune-related adverse events.

*General population frequencies are indicated by cited references.

#These two patients discontinued ICI because of AE.

Numbers in parentheses denote references listed in bibliography. A given patient may be included in more than one AE category.

XP patients developed AE/irAE with early onset (within 12 months after starting ICI) and delayed onset (arose later than 12 months after starting ICI (41)). The duration of these AE/irAE was acute (persisted less than 3 months) or chronic (persisted at least 3 months). For example, XP572BE developed AE/irAE with early onset and acute duration, which includes cough, elevated liver enzymes, abdominal pain, and hot flashes. XP495BE developed hypothalamic hypothyroidism that had a delayed onset and chronic duration (**Supplementary Figure S3**).

AE/irAE most frequently experienced by XP patients were cutaneous AE/irAE (*n* = 6; 27%), fatigue (*n* = 5; 23%), and endocrinopathies (*n* = 4; 18%). Cutaneous AE/irAE consisted of skin hypopigmentation (vitiligoid depigmentation/vitiligo) (*n* = 2; 9%), inflammation (*n* = 1; 5%), rash (eczematous dermatitis, punctate and macular rash, and rash in sun-damaged skin) (*n* = 3; 14%), and pruritus (*n* = 2; 9%) which occurred within 0.25–13 months after beginning ICI. Patients experienced fatigue within 13 months after beginning ICI. Endocrinopathies consisted of hypothyroidism (*n* = 4; 18%), acute nontraumatic kidney injury (*n* = 1; 5%), and adrenal insufficiency (*n* = 1; 5%), which occurred 4–27 months after beginning ICI. Additional AE/irAE categories experienced by XP patients are noted in **Table 2**; **Supplementary Tables S1–S3**.

To assess whether XP patients are at greater risk of AE from ICI, we compared the frequency of AE/irAE in XP patients to general population patients treated with ICI. The frequency of any- grade AE within the general population was 78% (632 of 811 patients) (19). Severe, CTCAE Grades 3–5 AE were experienced by 18% (146 of 811) of general population patients (19). AE/irAE more commonly experienced by general population patients were fatigue (188 of 811; 23%) (19) and cutaneous AE/irAE (2,171 of 8,637 patients; 25%) (42). Although numbers of XP patients are small, the type and frequencies of AE/irAE appear to be similar to general population (**Table 2**) (43–46).

### 4.4 Tumor mutational burden in five XP patients

XP patients have germline mutations in genes involved in nucleotide excision repair pathway and translesion synthesis (*POLH*) genes. Patients with germline mutations in another type of DNA- repair disorder (deficient mismatch-repair—Lynch syndrome) have a high frequency of colon cancers. These tumors were shown to have large numbers of mutations and responded well to ICI (47, 48). Tumors can also develop spontaneous mismatch-repair deficiency. Pathological or complete response was observed in 100% of mismatch-repair deficient colon tumors (20 of 20) (48) and rectal tumors (12 of 12) (47) in both germline or somatic mismatch-repair deficient patients.

We collected available information on tumor mutational burden in XP patients treated with ICI (**Table 3**). Within the NIH, UK, and literature review cohorts, there were five XP patients who had cancers analyzed for tumor mutational burden. Overall, the tumors from the five XP patients showed higher mutation frequencies than median frequencies from cancers in the general population (**Table 3**). All five XP patients had target cancers that responded to ICI treatment (**Table 1**). However, two of the five patients had additional cancers that did not respond to ICI despite having a high mutational Burden (22, 31) (**Table 3**). These two patients with more than one cancer were treated with ICI and one cancer responded while the other did not. This implies that the response is in part dependent on factors in the different cancers. Since the second cancers had high numbers of mutations, this suggests that there are other important factors than tumor mutational burden in predicting positive outcomes from ICI treatment.

**Table 3:** Tumors from five XP patients showed higher tumor mutational burden than tumors in the general population.

CANCER TYPE	XP PATIENTS [mutations/Mb]	GENERAL POPULATION [median tumor mutations/Mb](54)
Basal cell carcinoma	267-330(27), 227(25)^	47.3
Squamous cell carcinoma	53-460(22)*^b^, 100(25)	45.2
Non-small cell lung cancer	62* (XP376BE)	8.1
Angiosarcoma	248(31)*	3.3

XP, xeroderma pigmentosum; Mb, megabase; ICI, immune checkpoint inhibitor.

Numbers in parentheses denote references listed in bibliography.

*Tumors responded to ICI treatment.

^^^Basal cell carcinoma progressed during ICI treatment for angiosarcoma.

^b^Corneal squamous cell carcinoma progressed during ICI while squamous cell carcinomas on eyelid, conjunctiva, preauricular masses, lymph node, and bone regressed.

## 5 Discussion

This international retrospective study includes XP patients from the NIH, UK, and literature representing a relatively large cohort of this extremely rare cancer-prone disease. Seven XP patients did not respond to treatment with cytokines, chemotherapy, radiotherapy, or targeted antibody therapy before beginning ICI treatment. In contrast, all XP patients (22 of 22; 100%) showed regression or remission of target cancers in response to ICI (**Table 1**). The response rate in XP patients appears to be as least as high as in the general population where only 15%–60% of patients respond to ICI treatment (49). Similarly, all patients with germline or somatic mutations in another type of DNA-repair disorder (mismatch-repair) had a 100% response to ICI for colon (20 of 20) (48) and rectal cancers (12 of 12) (47). This high response rate may be due to increased mutation burden in cancers with deficient DNA repair (**Table 3**) (50).

Since deficient DNA repair in XP patients can lead to increased numbers of mutations in cancer and non-cancer cells/tissues (2–4), we were concerned that ICI may lead to increased severe AE. From our study, we found that XP patients experienced similar frequencies and types of AE as seen in the general population, suggesting ICI can be well tolerated in XP patients (**Table 2**).

Although target cancers of all XP patients responded to ICI, two patients had additional tumors that did not respond to ICI despite high mutational burden (22, 31). A proportion of general population patients with high mutational burden also did not respond to ICI treatment (11–13). Phase I clinical trial showed only 45.3% (63 of 139) of patients with highly mutated stage IV or recurrent non-small cell lung cancer had partial or complete response to treatment with nivolumab plus iplimumab (11). Within the XP and general population, multiple tumors did not respond to ICI despite high mutational burden, suggesting the existence of other factors contributing to different treatment outcomes. Cancers unresponsive to ICI may be due to mechanisms including loss of T-cell function and development of escape mutation variants in cancer cells (9, 51). New skin cancers (melanoma, BCC, and SCC) developed during and after ICI treatment in eight of 22 XP patients (**Table 1**). Studies showed conflicting findings on whether ICI reduce or increase the incidence of second primary cancers in the general population (52–54).

This study has limitations including the small sample size of the XP population and its retrospective nature. Within the literature review, there was possible bias in reporting positive clinical responses to ICI and limited duration of follow-up in case reports of several XP patients. When comparing the efficacy and AE of ICI between XP patients and the general population, we could not control for differences in treatment (e.g., drug type and dosage).

In conclusion, XP patients with metastatic or unresectable cancers demonstrated positive and well-tolerated responses to ICI. However, two XP patients had additional cancers that did not respond to ICI, two XP patients had cancer recurrence or progression after initial response and eight XP patients developed new skin cancers during or after ICI treatment. Future studies assessing levels of immunogenicity can provide insights into the lack of response to ICI in these cancers.

## Data availability statement

The original contributions presented in the study are included in the article/**Supplementary Material**. Further inquiries can be directed to the corresponding author.

## Ethics statement

The studies involving humans were approved by National Institutes of Health, National Cancer Institute Institutional Review Board. The studies were conducted in accordance with the local legislation and institutional requirements. The participants provided their written informed consent to participate in this study. Written informed consent was obtained from the individual(s) for the publication of any potentially identifiable images or data included in this article.

## Author contributions

EF: Data curation, Investigation, Methodology, Visualization, Writing – original draft, Writing – review & editing. DT: Conceptualization, Data curation, Investigation, Methodology, Supervision, Writing – review & editing. SK: Data curation, Investigation, Methodology, Validation, Writing – review & editing. SM: Data curation, Investigation, Methodology, Writing – review & editing. HF: Data curation, Investigation, Methodology, Writing – review & editing. RS: Funding acquisition, Project administration, Supervision, Writing – review & editing. JD: Conceptualization, Data curation, Formal Analysis, Investigation, Methodology, Supervision, Writing – review & editing. KK: Conceptualization, Data curation, Funding acquisition, Investigation, Methodology, Project administration, Resources, Supervision, Validation, Visualization, Writing – original draft, Writing – review & editing.
