# Supplementary material for: Retrospective study of efficacy and adverse events of immune checkpoint inhibitors in 22 xeroderma pigmentosum patients with metastatic or unresectable cancers
Source: Front Oncol. 2023 Oct 25;13:1282823. doi: 10.3389/fonc.2023.1282823 (PMC10634243; doi:10.3389/fonc.2023.1282823)
Supplement: Supplementary file 1 [file Presentation_1.pdf]

Retrospective study of efficacy and adverse events of immune checkpoint inhibitors in 22 xeroderma pigmentosum patients with metastatic or unresectable cancers

Elvelyn Fernandez[1], Deborah Tamura[1], Sikandar G. Khan[1], Sophie Momen[2], Hiva Fassihi[2], Robert Sarkany[2], John J. DiGiovanna[1], Kenneth H. Kraemer[1*]

Supplemental Figures
Figure S1 – NIH Patient Timelines
Figure S2 – UK Patient Timeline
Figure S3 - Duration and Onset of Adverse Events
Supplemental Tables
Supplemental table 1. NIH cohort of 6 XP patients treated with ICI
Supplemental table 2. UK National XP Clinic cohort of 3 XP patients treated with ICI
Supplemental table 3. Reported cases of 13 XP patients treated with ICI

Figure S1 – NIH Patient Timelines

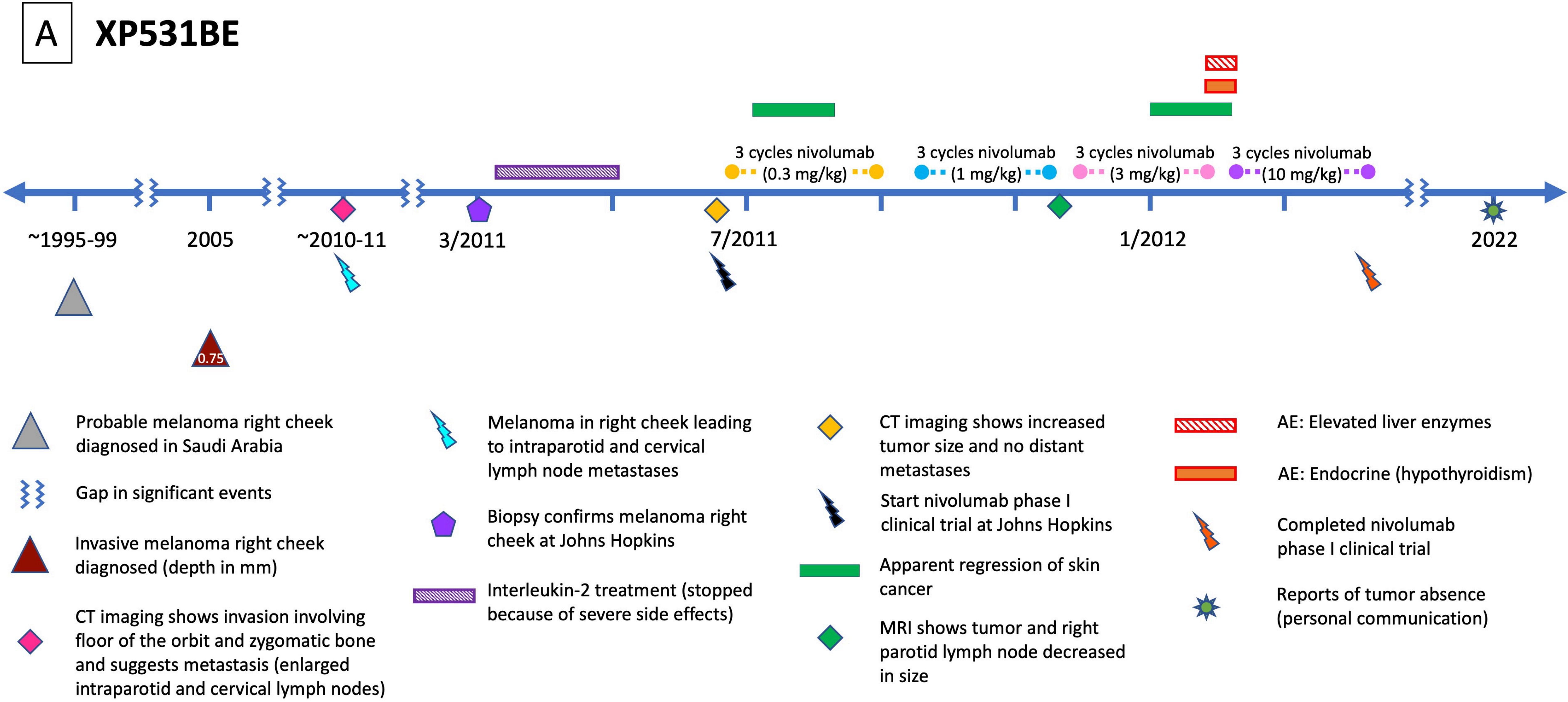

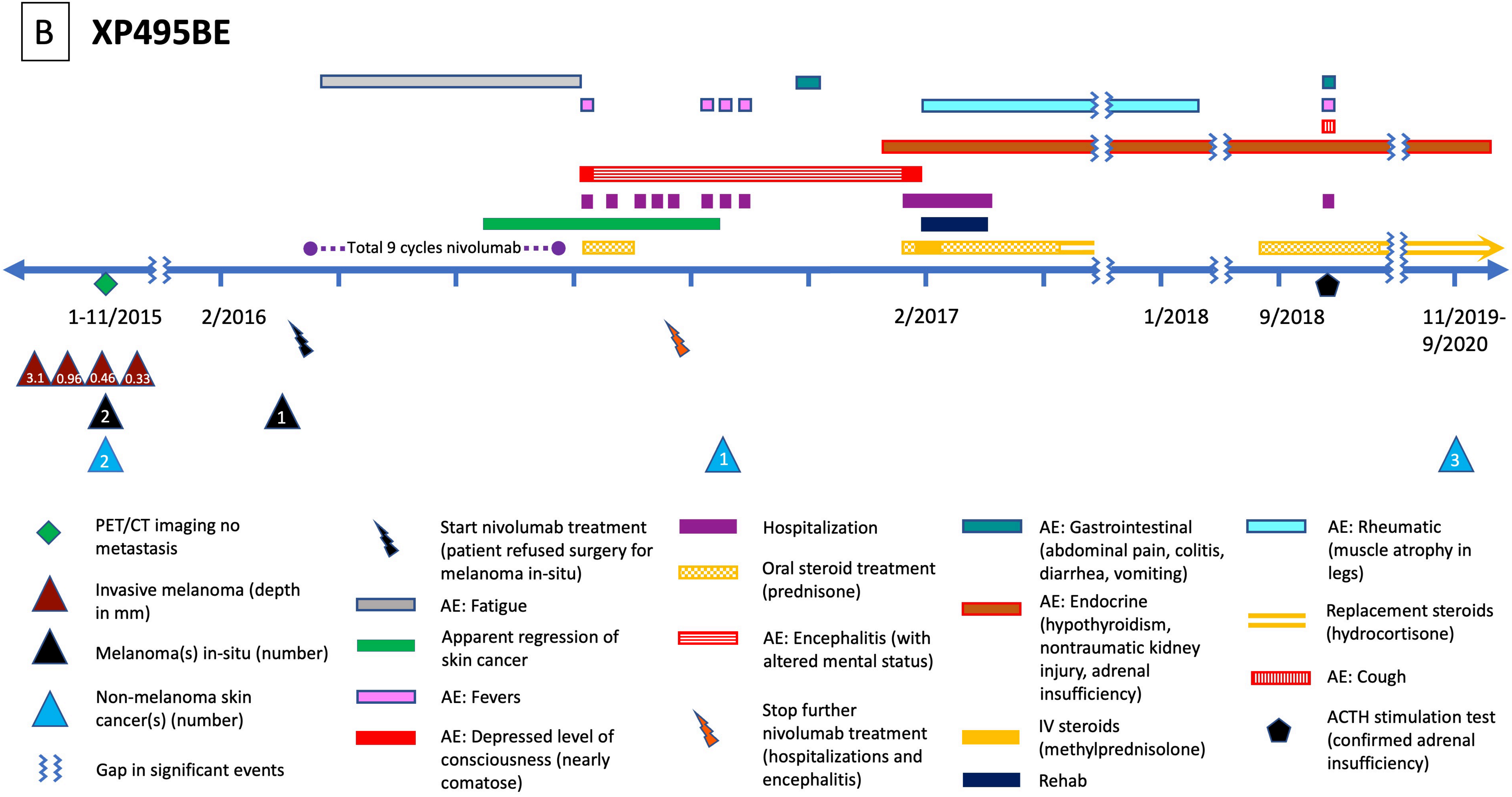
B
XP495BE
Total 9 cycles nivolumab
1-11/2015
2/2016
2/2017
1/2018
9/2018
11/2019-9/2020
3.1
0.96
0.46
0.33
2
1
2
1
3
PET/CT imaging no metastasis
Invasive melanoma (depth in mm)
Melanoma(s) in-situ (number)
Non-melanoma skin cancer(s) (number)
Gap in significant events
Start nivolumab treatment (patient refused surgery for melanoma in-situ)
AE: Fatigue
Apparent regression of skin cancer
AE: Fevers
AE: Depressed level of consciousness (nearly comatose)
Hospitalization
Oral steroid treatment (prednisone)
AE: Encephalitis (with altered mental status)
Stop further nivolumab treatment (hospitalizations and encephalitis)
AE: Gastrointestinal (abdominal pain, colitis, diarrhea, vomiting)
AE: Endocrine (hypothyroidism, nontraumatic kidney injury, adrenal insufficiency)
IV steroids (methylprednisolone)
Rehab
AE: Rheumatic (muscle atrophy in legs)
Replacement steroids (hydrocortisone)
AE: Cough
ACTH stimulation test (confirmed adrenal insufficiency)

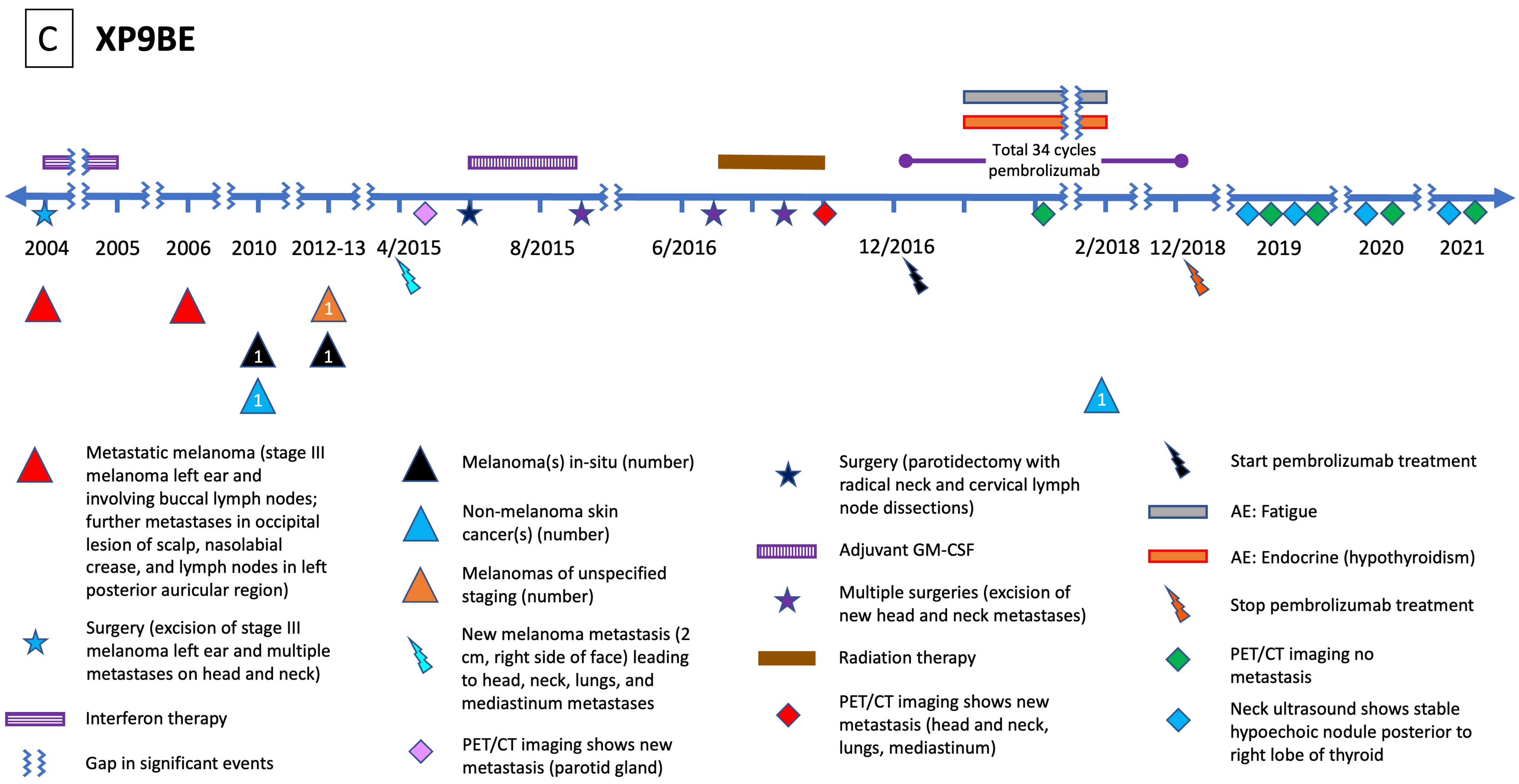
C
XP9BE
Total 34 cycles pembrolizumab
2004
2005
2006
2010
2012-13
4/2015
8/2015
6/2016
12/2016
2/2018
12/2018
2019
2020
2021
1
1
1
1
1
Metastatic melanoma (stage III melanoma left ear and involving buccal lymph nodes; further metastases in occipital lesion of scalp, nasolabial crease, and lymph nodes in left posterior auricular region)
Surgery (excision of stage III melanoma left ear and multiple metastases on head and neck)
Interferon therapy
Gap in significant events
Melanoma(s) in-situ (number)
Non-melanoma skin cancer(s) (number)
Melanomas of unspecified staging (number)
New melanoma metastasis (2 cm, right side of face) leading to head, neck, lungs, and mediastinum metastases
PET/CT imaging shows new metastasis (parotid gland)
Surgery (parotidectomy with radical neck and cervical lymph node dissections)
Adjuvant GM-CSF
Multiple surgeries (excision of new head and neck metastases)
Radiation therapy
PET/CT imaging shows new metastasis (head and neck, lungs, mediastinum)
Start pembrolizumab treatment
AE: Fatigue
AE: Endocrine (hypothyroidism)
Stop pembrolizumab treatment
PET/CT imaging no metastasis
Neck ultrasound shows stable hypoechoic nodule posterior to right lobe of thyroid

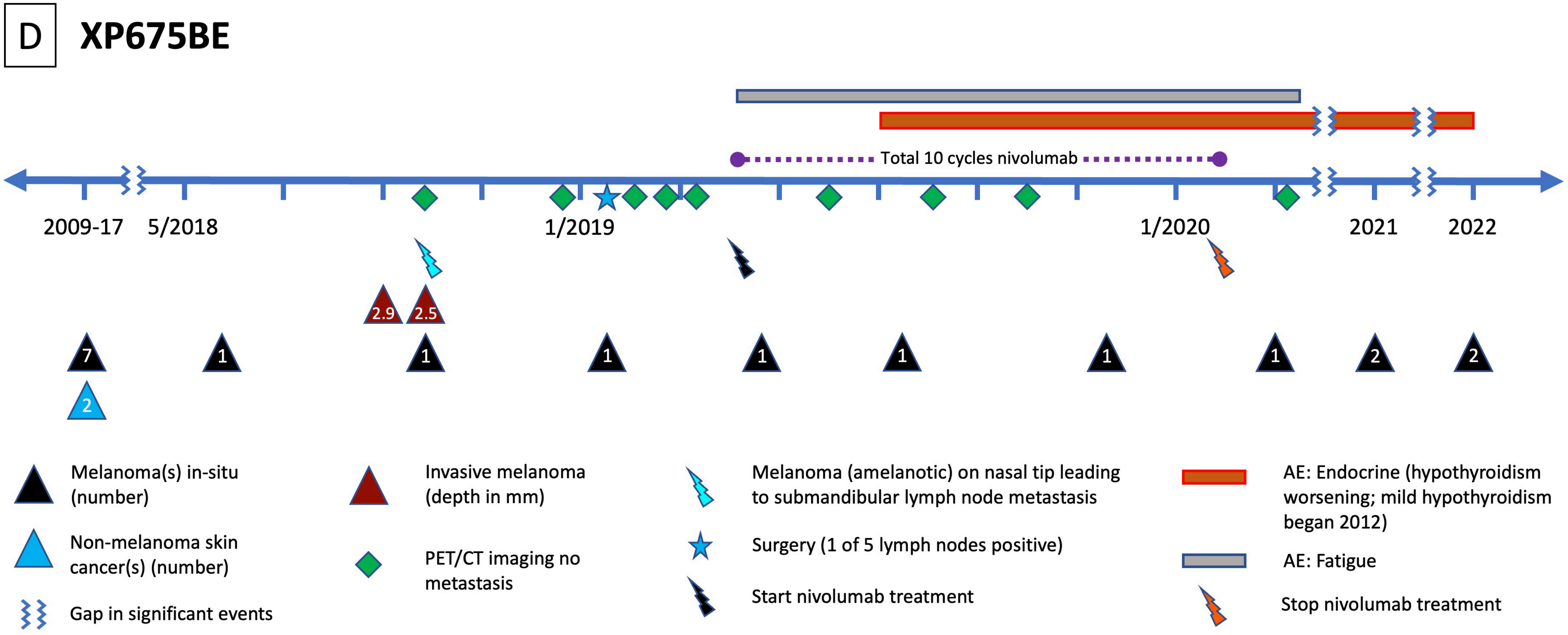
D
XP675BE
Total 10 cycles nivolumab
2009-17
5/2018
1/2019
1/2020
2021
2022
2.9
2.5
7
2
1
1
1
1
1
1
1
2
2
Melanoma(s) in-situ (number)
Non-melanoma skin cancer(s) (number)
Gap in significant events
Invasive melanoma (depth in mm)
PET/CT imaging no metastasis
Melanoma (amelanotic) on nasal tip leading to submandibular lymph node metastasis
Surgery (1 of 5 lymph nodes positive)
Start nivolumab treatment
AE: Endocrine (hypothyroidism worsening; mild hypothyroidism began 2012)
AE: Fatigue
Stop nivolumab treatment

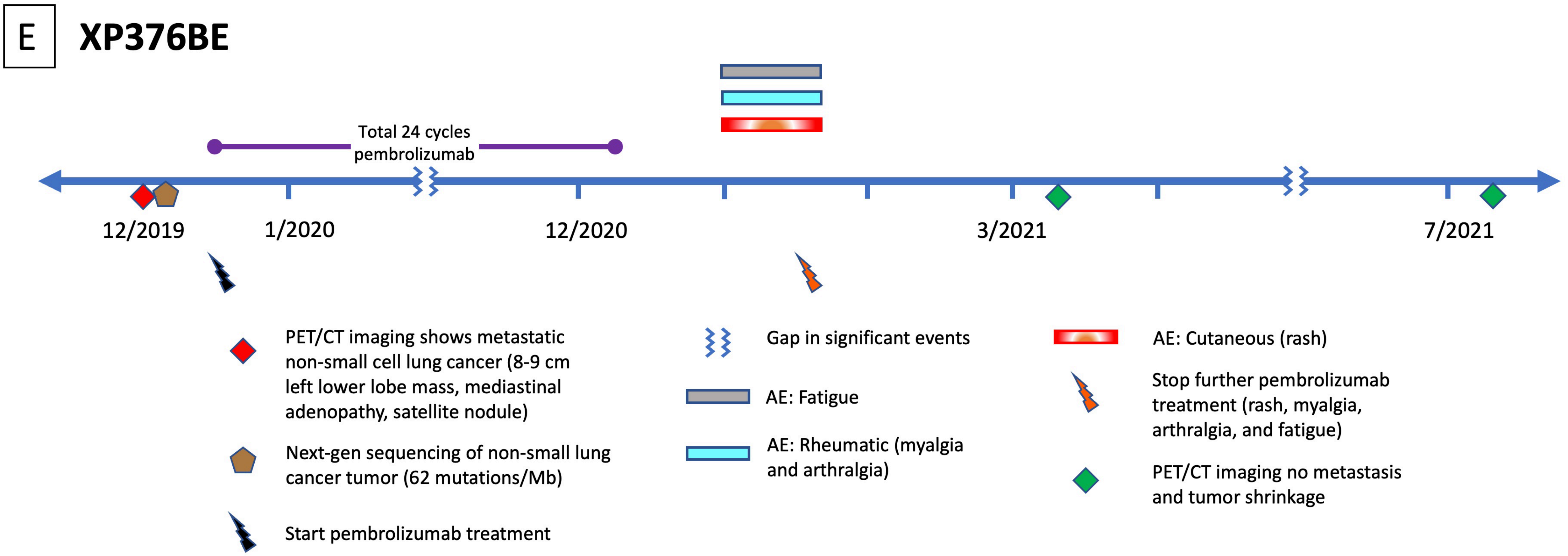
E
XP376BE
Total 24 cycles pembrolizumab
12/2019
1/2020
12/2020
3/2021
7/2021
PET/CT imaging shows metastatic non-small cell lung cancer (8-9 cm left lower lobe mass, mediastinal adenopathy, satellite nodule)
Next-gen sequencing of non-small lung cancer tumor (62 mutations/Mb)
Start pembrolizumab treatment
Gap in significant events
AE: Fatigue
AE: Rheumatic (myalgia and arthralgia)
AE: Cutaneous (rash)
Stop further pembrolizumab treatment (rash, myalgia, arthralgia, and fatigue)
PET/CT imaging no metastasis and tumor shrinkage

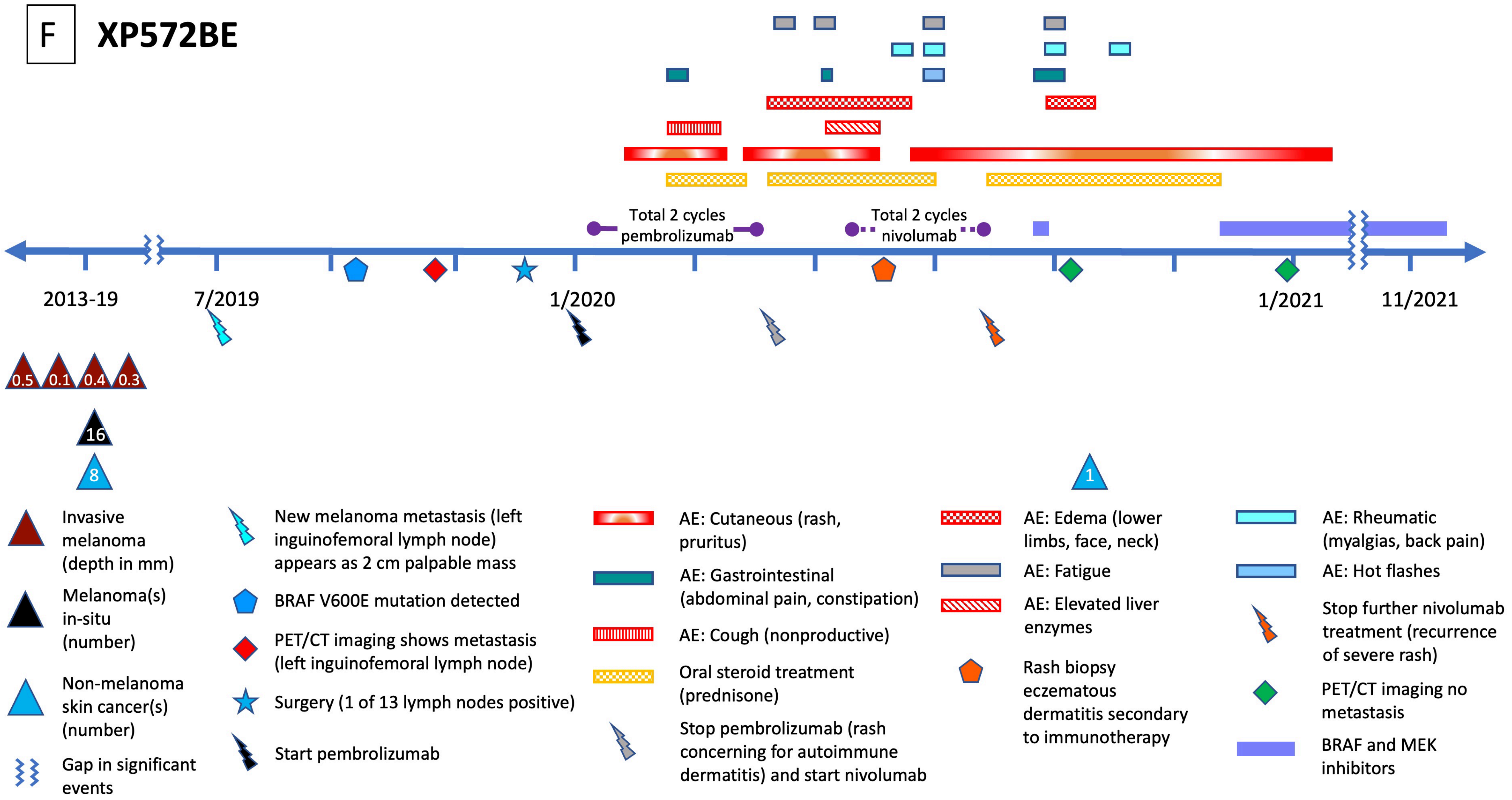
F
XP572BE
Total 2 cycles pembrolizumab
Total 2 cycles nivolumab
2013-19
7/2019
1/2020
1/2021
11/2021
0.5
0.1
0.4
0.3
16
8
1
Invasive melanoma (depth in mm)
Melanoma(s) in-situ (number)
Non-melanoma skin cancer(s) (number)
Gap in significant events
New melanoma metastasis (left inguinofemoral lymph node) appears as 2 cm palpable mass
BRAF V600E mutation detected
PET/CT imaging shows metastasis (left inguinofemoral lymph node)
Surgery (1 of 13 lymph nodes positive)
Start pembrolizumab
AE: Cutaneous (rash, pruritus)
AE: Gastrointestinal (abdominal pain, constipation)
AE: Cough (nonproductive)
Oral steroid treatment (prednisone)
Stop pembrolizumab (rash concerning for autoimmune dermatitis) and start nivolumab
AE: Edema (lower limbs, face, neck)
AE: Fatigue
AE: Elevated liver enzymes
Rash biopsy eczematous dermatitis secondary to immunotherapy
AE: Rheumatic (myalgias, back pain)
AE: Hot flashes
Stop further nivolumab treatment (recurrence of severe rash)
PET/CT imaging no metastasis
BRAF and MEK inhibitors

Figure S2 – UK Patient Timeline

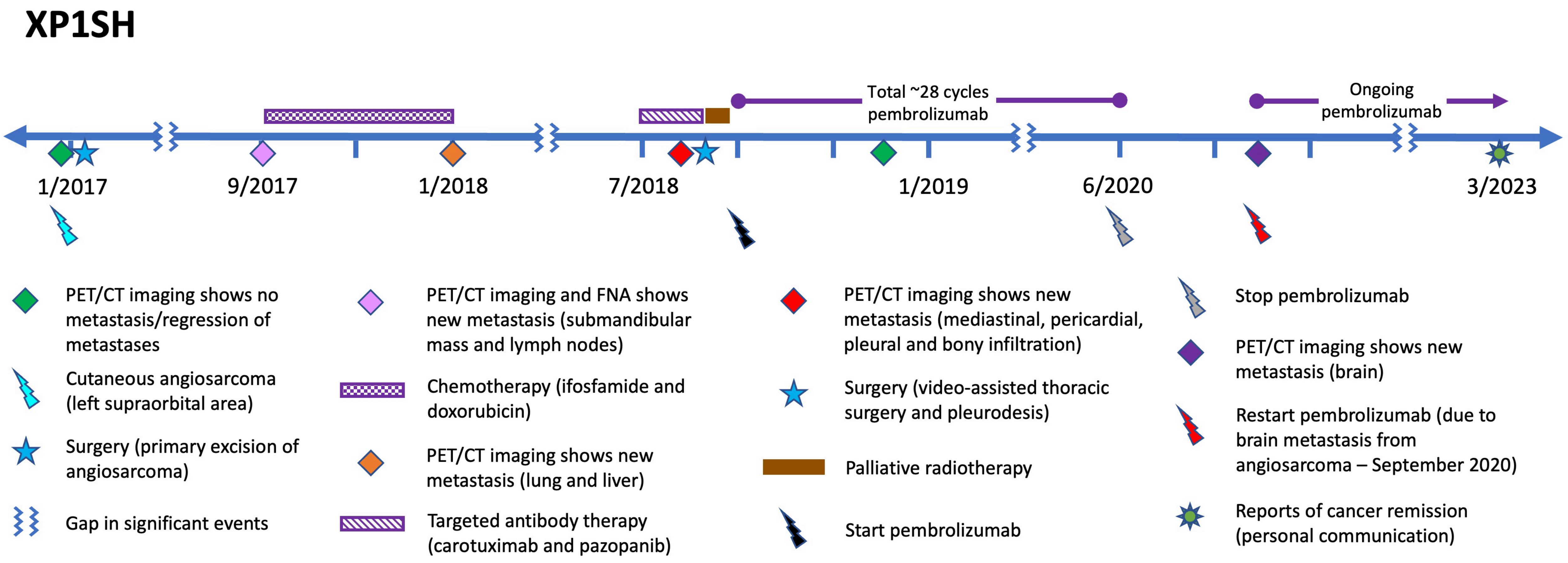

Figure S3 - Duration and Onset of Adverse Events

	Early Onset (AE/irAE arising $\leq$12 mo after 1st ICI cycle) (10 patients)	Delayed Onset (AE/irAE arising >12 mo after 1st ICI cycle) (2 patients)
Acute Duration (AE/irAE persisting <3 mo) (7 patients)	1. XP531BE: **endocrine** (*hypothyroidism*), **elevated liver enzymes**<br>2. XP495BE: **fatigue, fever, depressed level of consciousness** *(nearly comatose),* **gastrointestinal** (*colitis/GI infection, diarrhea*, *vomiting,* and *abdominal pain),* **endocrine** (*acute nontraumatic kidney injury*)<br>3. XP9BE**: fatigue, endocrine** (*subclinical hypothyroidism*)<br>4. XP572BE: **cough**, **elevated liver enzymes, gastrointestinal** (*abdominal pain)*, **hot flashes**<br>5. Female XP-C patient, age 49 (Scheer et al.): **cutaneous** (*rash in sun-damaged skin*)<br>6. Male XP-E patient, age 51 (Hauschild et al.): **cutaneous** (*inflammation in sun-damaged skin, pruritus*)<br>7. Female XP patient, age 19 (Rubatto et al.): **gastrointestinal** (*diarrhea*)	1. XP495BE: **Cough**
Chronic duration (AE/irAE persisting $\geq$3 mo) (6 patients)	1. XP495BE: **Encephalitis** *(with altered mental status)*, **rhematic** (*muscle atrophy in legs*), **endocrine** (*adrenal insufficiency)*<br>2. XP675BE: **fatigue**, **endocrine** (*hypothyroidism*)<br>3. XP572BE: **cutaneous** (*rash*, *pruritus*), **fatigue**, **edema** (*lower limbs, face, neck*), **rheumatic** (*myalgia*, *back pain*), **gastrointestinal** (*constipation*)<br>4. XP136LO: **cutaneous** (*vitiligo*)<br>5. Male XP-C patient, age 17 (Salomon et al.): **cutaneous** (*vitiligoid depigmentation)*	1. XP495BE: **Endocrine** (*hypothalamic hypothyroidism*)<br>2. XP376BE: **Cutaneous** (*punctate and macular rash*), **rheumatic** (*myalgia and arthralgia)*, **fatigue**

Supplemental table 1. NIH cohort of 6 XP patients treated with ICI.

AGE/SEX XP NUMBER	XP GROUP (GENE)	TARGET CANCER (METASTASES)	ADDITIONAL CANCER HISTORY	RECEIVED TREATMENT BEFORE ICI?	ICI TREATMENT [YEAR] (DOSAGE)	ADVERSE EVENTS^	TARGET CANCER RESPONSE/ NEW CANCERS DETECTED DURING OR AFTER ICI?	CURRENT STATUS*
34/M XP531BE	XP-D (*ERCC2)*	Metastatic melanoma from right cheek (intraparotid and cervical lymph node)	Multiple melanomas treated surgically[a]	YES (interleukin-2)[b]	Phase I clinical trial of nivolumab [2011-2012] (0.3-10 mg/kg/mo, 10 mo, 12 cycles)	Grade 1: hypothyroidism, elevated liver enzymes	REMISSION/ NO NEW CANCERS	Target cancer remission persisting 119 mo after last ICI cycle.
63/M XP495BE	XP-C (*XPC)*	Multiple primary melanomas (without metastasis)	Multiple skin cancers (melanoma, BCC, SCC) treated surgically[c]	NO	nivolumab [2016] (260-271 mg/2 weeks, 4 mo, 9 cycles)	**Grade 3-4:** encephalitis (with altered mental status), depressed level of consciousness (nearly comatose), colitis (GI infection)[d]<br>Grade 1-2: fatigue, hypothyroidism, adrenal insufficiency, vomiting, abdominal pain, cough<br>Unknown grade: fever, acute nontraumatic kidney injury, muscle atrophy, diarrhea	REGRESSION/ NEW CANCERS DETECTED	Target cancer regression persisting 61 mo after last ICI cycle.<br>3 BCCs and 1 SCC occurred 3-50 mo after last cycle.<br>Taking medication to treat adrenal insufficiency and hypothyroidism.
57/M XP9BE	XP-C (*XPC)*	Metastatic melanoma from right side of face (head, neck, lungs, and mediastinum)	Multiple skin cancers (melanoma, BCC, SCC) treated surgically	YES (interferon, adjuvant GM-CSF, radiation, and surgery)[e]	pembrolizumab [2016-2018] (24 mo, 34 cycles)	Grade 1: fatigue, hypothyroidism (subclinical)	REMISSION/ NEW CANCER DETECTED	Target cancer remission persisting 60 mo after first PET/CT no evidence of metastasis.<br>1 cSCC occurred during ICI treatment.
58/F XP675BE	XP-C (XPC)	Metastatic amelanotic melanoma from nasal tip (submandibular lymph node)	Multiple skin cancers (melanoma, BCC, SCC) treated surgically, cancer in ovary and uterus[f]	YES (surgery)	nivolumab [2019-2020] (480 mg/cycle, 10 mo, 10 cycles)	Grade 2: fatigue, hypothyroidism	REMISSION/ NEW CANCERS DETECTED	Target cancer remission persisting 45 mo after first PET/CT no evidence of metastasis.<br>3 melanomas in-situ occurred during ICI treatment.<br>5 melanomas in-situ occurred 2-32 mo after last ICI cycle.
59/F XP376BE	XP-C (*XPC)*	Metastatic NSCLC from left lower lobe mass (mediastinal adenopathy and satellite nodule in left upper lobe)	Multiple skin cancers (melanoma, BCC, SCC) treated surgically[g]	NO	pembrolizumab [2019-2020] (400 mg/cycle, 12 mo, 24 cycles)	Grade 1-2: rash (punctate & macular), myalgia, arthralgia	REMISSION/ NO NEW CANCERS	Target cancer remission persisting 20 mo after first PET/CT no evidence of metastasis.
35/F XP572BE	XP-C (*XPC)*	Metastatic melanoma with unknown primary (left inguinofemoral lymph node)	Multiple skin cancers (melanoma, BCC, SCC) treated surgically[h]	YES (surgery)	Pembrolizumab [2020] (1.5 mo, 2 cycles) and nivolumab (1 mo, 2 cycles)	**Grade 3:** rash (eczematous dermatitis, Fig 2), pruritus, elevated liver enzymes<br>Grade 1-2: fatigue, hot flashes, edema (lower limbs, face, neck), myalgia, back pain, abdominal pain, constipation, cough (nonproductive)	REMISSION/ NEW CANCER DETECTED	Target cancer remission persisting 13 mo after first PET/CT no evidence of metastasis.<br>1 BCC occurred 3 mo after last ICI cycle.<br>Due to AE, ICI stopped and began 12 mo BRAF/MEK inhibitors.

NIH, National Institutes of Health; XP, xeroderma pigmentosum; ICI, immune checkpoint inhibitors; NSCLC, non-small cell lung cancer; BCC, basal cell carcinoma; SCC, squamous cell carcinoma; cSCC, cutaneous squamous cell carcinoma; GM-CSF, granulocyte macrophage colony-stimulating factor; mo, months; GI, gastrointestinal; PET, positron emission tomography; CT, computed tomography; AE, adverse events

^The Common Terminology for Adverse Events (CTCAE) v5.0 was used to grade AE.

*Current status was assessed at day of last follow-up with each patient.

[a]The patient also has hypertension and Type 2 diabetes.

[b]Before nivolumab treatment, the patient's metastatic melanoma was unsuccessfully treated with interleukin-2 (Supplemental figure 1A).

[c]Patient also has hypertension.

[d]GI infection and subsequent colitis was treated with oral antibiotics.

[e]Twelve years before pembrolizumab treatment, the patient developed a stage III melanoma from his left ear with lymph node, scalp, and nasolabial crease metastases. He was treated with surgery and interferon. Ten years later, he developed a 2 cm melanoma on his face with a right parotid gland metastasis. He was unsuccessfully treated with surgery and GM-CSF. Further metastases involving the head, neck, lungs, and mediastinum were treated with surgery, radiation therapy (31 rounds), and pembrolizumab (Supplemental Figure 1C).

[f]The patient reported that 24 years before nivolumab treatment for metastatic melanoma, she had cancer of unspecified type in one ovary and in the uterus treated with a total abdominal hysterectomy and bilateral oophorectomy. The patient also has a history of hypothyroidism treated with chronic Synthroid for at least 8 years before nivolumab treatment.

[g]The patient has a history of smoking (0.5 packs/day for 30 years) and emphysema.

[h]The patient has additional conditions of osteoporosis and premature menopause due to XP.

Supplemental table 2. UK National XP Clinic cohort of 3 XP patients treated with ICI.

AGE/SEX XP NUMBER	XP GROUP (GENE)	TARGET CANCER (METASTASES)	ADDITIONAL CANCER HISTORY	RECEIVED TREATMENT BEFORE ICI?	ICI TREATMENT [YEAR] (DOSAGE)	ADVERSE EVENTS^	TARGET CANCER RESPONSE/ NEW CANCERS DETECTED DURING OR AFTER ICI?	CURRENT STATUS*
32/M[31, 36] XP1SH	XP-C (*XPC*)	Metastatic cutaneous angiosarcoma from left supraorbital area (submandibular mass and lymph nodes, lung, liver, mediastinum, pericardium, pleura, and bone) (Metastasis to brain 3 mo after completion of first course of ICI)	Multiple non-melanoma skin cancers (BCC, SCC) (unspecified treatment)	YES (chemotherapy - ifosfamide & doxorubicin) (targeted antibody therapy - carotuximab & angiogenesis inhibitor pazopanib) (palliative radiotherapy)[a]	pembrolizumab [2018-present] (200 mg/3 weeks, 21 mo, 28 cycles, then 3-month break; resumed ICI for 30 mo as of March 2023, ongoing treatment)	—	REMISSION/ NO NEW CANCERS	During a 3-month break from ICI, the patient developed a brain metastasis from angiosarcoma. Continued target cancer remission 30 mo after resuming ICI. Ongoing treatment as of March 2023.
62/M XP136LO	XP variant (*POLH*)	Metastatic melanoma with unknown primary (mid-upper back and lung)	Multiple skin cancers (melanoma, BCC, SCC) (unspecified treatment)	YES (surgery)	nivolumab [2018-2020] (480 mg, 18 mo, 18 cycles)	Grade 1: skin hypopigmentation (vitiligo secondary to nivolumab)	REMISSION/ NEW CANCER DETECTED	Target cancer remission persisting 35 mo after last ICI cycle. 1 invasive melanoma (pT2a) occurred 25 mo after last ICI cycle.
33/M XP124NR	XP-C (*XPC*)	Metastatic melanoma with unknown primary (parotid gland)	Multiple non-melanoma skin cancers (BCC, SCC) (some treated surgically)	NO	pembrolizumab [2019-2021] (400 mg/6 weeks, 24 mo, 16 cycles)	—	REMISSION/ NO NEW CANCERS	Target cancer remission persisting 36 mo after first PET/CT no evidence of metastasis.

UK, United Kingdom; XP, xeroderma pigmentosum; ICI, immune checkpoint inhibitors; BCC, basal cell carcinoma; SCC, squamous cell carcinoma; mo, months; PET, positron emission tomography; CT, computed tomography

^The Common Terminology for Adverse Events (CTCAE) v5.0 was used to grade adverse events.

*Current status was assessed at day of last follow-up with each patient or healthcare provider.

[a]Before pembrolizumab treatment, the patient developed a right submandibular metastasis from angiosarcoma and was treated with ifosfamide and doxorubicin. After 6 cycles, he developed lung and liver metastases and was then treated with carotuximab and pazopanib for 1 month. He developed further mediastinal, pericardial, pleural, liver, and bone metastases treated with video-assisted thoracic surgery and pleurodeses. Mandibular tumor deposit was treated with palliative radiotherapy (36 Gy, 12 fractions) (Supplemental Figure 2).

Supplemental table 3. Reported cases of 13 XP patients treated with ICI.

AGE/ SEX	XP GROUP (GENE)	TARGET CANCER	ADDITIONAL CANCER HISTORY	RECEIVED TREATMENT BEFORE ICI?	ICI TREATMENT (DOSAGE)	ADVERSE EVENTS^	TARGET CANCER RESPONSE/ NEW CANCERS DETECTED DURING OR AFTER ICI?	CURRENT STATUS*
6/ F[20]	XP-C (*XPC)*	Sarcomatoid carcinoma of the scalp (without metastasis)	Multiple non-melanoma skin cancers (BCC, SCC) treated surgically	YES (chemotherapy - 5FU & cisplatin) (surgery)[a]	nivolumab (3 mg/kg/2 weeks then monthly, 13 mo, 16 cycles)[b]	—	REGRESSION/ NEW CANCERS DETECTED	Continued target cancer regression 13 mo after first ICI cycle. Treatment ongoing as of publication. Several SCCs and 2 large skin melanomas occurred during ICI treatment.
6/M[21]	XP-C (*XPC*)	Metastatic cSCC from nose (regional lymph node) (Recurrence on the neck 18 mo after completion of first course of ICI treatment)	Multiple non-melanoma skin cancers (SCC) treated surgically	YES (5FU, cisplatin, surgery)[c]	nivolumab (4 mo, 6 cycles for first occurrence; 15 mo, ~21 cycles for second occurrence)[d]	—	REMISSION/ NO NEW CANCERS	New superficial lesions appeared on scalp, tongue, and right auricle after first ICI treatment (4 mo) and treated with 5FU. SCC tumor recurrred 18 mo after completion of first course of ICI treatment. Target cancer remission after second course of ICI persisting at time of publication (unknown dates for last cycle of ICI or PET/CT imaging no evidence of metastasis).
7/F[22]	XP-C (*XPC*)	Metastatic SCC from left lower eyelid, right conjunctiva, right cornea, and preauricular masses (right parotid lymph node and bone)	Multiple skin cancers (unspecified cancer type) treated surgically	NO	pembrolizumab (2 mg/kg/3 weeks, 24 mo, 9 cycles)	—	REGRESSION/ NO NEW CANCERS	Continued cutaneous and mucous membrane SCC regression 24 mo after first ICI cycle. Corneal SCC did not respond to ICI treatment and was instead treated with 5FU.[e] Treatment ongoing as of publication.
17/ M[23]	XP-C (*XPC*)	Metastatic melanoma with unspecified primary (liver and lung)	Multiple skin cancers (melanoma, BCC, SCC) treated surgically[f]	NO	pembrolizumab (2 mg/kg/3 weeks, 9 mo, 12 cycles)	Vitiligoid depigmentation mainly on UV-exposed areas	REGRESSION/ NO NEW CANCERS	Continued target cancer regression 18 mo after first ICI cycle.[g] Treatment ongoing as of publication.
18/ F[24]	XP-C (*XPC*)	Unresectable ocular SCC involving limbus of right eye (without metastasis)	Multiple non-melanoma skin cancers (cSCC, BCC)[h]	NO	pembrolizumab (2 mg/kg/3 weeks, ~12 mo, ~17 cycles)	—	REMISSION/ NEW CANCER DETECTED	Target cancer remission persisting 14 mo after last ICI cycle. 1 cSCC occurred during ICI treatment.
29/ M[25]	XP-C (*XPC*)	Cutaneous angiosarcoma of the left inner canthus (without metastasis) Side target: BCC of the right face	Multiple non-melanoma skin cancers (SCC) (unspecified treatment)	YES (surgery, chemotherapy)[i]	nivolumab (18 mo, 34 cycles)	—	REMISSION/ NO NEW CANCERS	Angiosarcoma remission persisting 41 mo after last ICI cycle. BCC did not respond to ICI treatment and was instead treated with surgery and vismodegib.
49/ F[26]	XP-C (*XPC*)	Metastatic melanoma from right upper arm (left shoulder subcutaneous)	Multiple skin cancers (melanoma, BCC, SCC) surgically treated, hormone receptor-positive invasive ductal breast carcinoma[j]	YES (surgery)	nivolumab (12 mo, 25 cycles)	Rash in sun-damaged skin areas healed spontaneously in 8-12 weeks	REMISSION/ NO NEW CANCERS	Target cancer remission persisting 36 mo after last ICI cycle.

19/ M[24]	XP-E (*DDB2)*	Unresectable cSCC involving the right nasal cavity and right orbit (without metastasis)	Multiple non-melanoma skin cancers (cSCC) (unspecified treatment)	NO	pembrolizumab (2 mg/kg/3 weeks, 18 mo, 25 cycles)	—	REGRESSION/ NO NEW CANCERS	Continued target cancer regression 18 mo after first ICI cycle.
51/ M[27]	XP-E (*DDB2)*	Metastatic melanoma with unspecified primary (lymph nodes, lung, and right infraorbital area)<br>Side target: Multiple non-melanoma skin cancers (mostly BCCs)	Multiple skin cancers (melanoma, SCC, BCC) and cutaneous angiosarcoma treated surgically	NO	pembrolizumab (2 mg/kg/3 weeks, 7 mo, 10 cycles)	Short-term inflammation in sun-damaged skin and itching	REGRESSION/ NO NEW CANCERS	Continued target cancer regression 7 mo after first ICI cycle.[k]<br>Disappearance of almost all multiple non-melanoma skin cancers 3 mo after first cycle.<br>Possibly ongoing treatment.
20/ F[24]	XP variant (*POLH)*	1) Metastatic melanoma (unspecified primary and metastatic sites)<br>2) SCC maxillary sinus occurred ~84 mo after first cycle of ipilimumab for melanoma	Multiple skin cancers (unspecified cancer type and treatment)	NO	1) Melanoma treated with ipilimumab (10 mg/kg/3 weeks, ~60 mo, ~81 cycles)<br>2) SCC treated with pembrolizumab (140 mg/mo, 31 mo, 31 cycles)	—	1) REMISSION<br>2) REGRESSION/ NEW CANCER DETECTED	1) Melanoma remission persisting ~60 mo after last cycle of ipilimumab.<br>2) SCC regression continued 31 mo after first pembrolizumab cycle. Subsequent progression of cancer led to pembrolizumab discontinuation.<br>1 BCC occurred during or after pembrolizumab treatment (unclear timing).
58/ M[28]	XP variant (*POLH)*	Metastatic SCC from right cervical mass (parotid gland and cervical lymph nodes)	Multiple skin cancers (melanoma, SCC) treated surgically[l]	NO	Pembrolizumab (200 mg/3 weeks, 15 mo, 19 cycles)	—	REGRESSION/ NO NEW CANCERS	Continued target cancer regression 15 mo after first ICI cycle.
19/ F[29]	—	Metastatic cSCC from right periorbital and nasal area (laterocervical lymph node and submandibular area)	Multiple non-melanoma skin cancers (BCC, cSCC) treated with surgery and vismodegib[m]	YES (proton beam radiotherapy)[n]	cemiplimab (350mg/3 weeks, 5 mo, 7 cycles)	Grade 1: diarrhea	REGRESSION/ NO NEW CANCERS	Continued target cancer regression 5 mo after first ICI cycle.<br>Possibly ongoing treatment at time of publication.
48/ F[30]	—	Metastatic cSCC with unspecified primary (supraclavicular, abdominal, and inguinal lymph nodes)	Multiple non-melanoma skin cancers (cSCC, BCC) treated surgically[o]	YES (surgery)	pembrolizumab (2 mg/kg/3 weeks, 2 mo, 3 cycles)	—	REGRESSION/ NO NEW CANCERS	Continued target cancer regression 2 mo after first ICI cycle.<br>Ongoing treatment at time of publication.

XP, xeroderma pigmentosum; ICI, immune checkpoint inhibitors; BCC, basal cell carcinoma; SCC, squamous cell carcinoma; cSCC, cutaneous squamous cell carcinoma; 5FU, 5-fluorouracil; mo, months; PET, positron emission tomography; CT, computed tomography; AE, adverse events

^The Common Terminology for Adverse Events (CTCAE) v5.0 was used to grade AE.

*Current status was assessed at the last follow-up recorded in each case report.

[a]Before nivolumab treatment, patient had incomplete resections of sarcomatoid carcinoma and two unsuccessful courses of chemotherapy (1/gm^2/day of 5FU and 100 mg/m^2/day of cisplatin).

[b]During nivolumab treatment, the patient developed an SCC on the scalp near the initial lesion. She was then treated with combined monthly nivolumab with cetuximab (250 mg/m^2, 3 cycles). Cetuximab therapy stopped after patient developed melanomas. Patient had periodic excisions of additional tumors.

[c]Before nivolumab treatment, the first SCC occurrence (primary tumor on the nose with metastases to the submandibular and preauricular lymph nodes) was treated with chemotherapy (cisplatin and 5FU). After 6 chemotherapy cycles, the primary tumor was excised. After 2 more chemotherapy cycles, progressive disease was noted (enlarged submandibular lymph node and new lesion on the right eyelid).

[d]The first course of nivolumab treatment (4 mo) was planned with concomitant radiotherapy treatment (66 Gy to the primary nasal mass, 60 Gy to preauricular LN, and 54 Gy to bilateral neck). The SCC recurred on the nape and was treated with paclitaxel (weekly 80mg/m^2, 5 mo) then nivolumab (15 mo).

[e]No additional tumor progression of the right cornea was noted after 1 month of local 5FU treatment.

[f]At 12 years old, the patient had enucleation of left eye for an SCC infiltrating the inner canthus and the eye. Four years before pembrolizumab treatment, the patient developed a nodular and ulcerated melanoma of the scalp (Breslow thickness of 3.3 mm) which was treated surgically. Sentinal lymph node biopsy showed no metastasis. The patient was then followed with abdominal and lymph node ultrasounds every 6 months.

[g]Before pembrolizumab treatment, the patient had numerous actinic keratoses, BCC, and SCC mostly on the face and limbs. After four cycles of pembrolizumab treatment, the patient had regression of cutaneous carcinomas and disappearance of many actinic keratoses.

[h]Within eight months before starting pembrolizumab treatment, the patient developed 10 new cSCCs (unspecified treatment). The patient also had multiple BCCs on the face (unspecified number and time of appearance). They did not respond to pembrolizumab and were instead treated surgically. One month after starting pembrolizumab treatment, the patient was treated with topical calcipotriol and 5FU to manage actinic keratoses.

[i]Before nivolumab treatment, the angiosarcoma was incompletely resected and treated unsuccessfully with chemotherapy. At the same time, a recurrent invasive BCC was excised. The BCC relapsed and was treated with vismodegib for one month until the angiosarcoma progressed. The angiosarcoma completely responded to nivolumab (15 mo), allowing for large surgical resection of the BCC then vismodegib treatment (complete response within 6 mo; discontinued after 24 mo due to diarrheas).

[j]Sixteen years before nivolumab treatment, the patient developed hormone receptor-positive invasive ductal breast carcinoma and was treated with surgery, chemotherapy, radiotherapy, and anti-hormonal therapy for five years.

[k]Complete response could not be verified due to patient refusal of control tumor biopsies.

[l]The patient also has a history of inflammatory bowel disease treated with methotrexate (which was stopped before treatment with pembrolizumab), stage IA melanoma of the forehead treated by wide excision, and a 20-year long history of recurrent, multiple cSCCs of the face treated with multiple iterative surgeries. No flare-up of inflammatory bowel disease during pembrolizumab treatment.

[m]At 18 years old, patient received vismodegib for advanced BCC and developed alopecia.

[n]Before cemiplimab treatment, proton beam radiotherapy (total dose of 59.4 Gy in 33 fractions for 2-3 mo) was performed on the target cancer and was well tolerated. Subsequent MRI showed stable disease. Fifteen months later, follow-up MRI showed laterocervical lymph node progression with a pathological submandibular lesion. Cemiplimab treatment was then initiated.

[o]Around five years before pembrolizumab treatment, the patient developed a poorly differentiated cSCC with invasion into the lymphatic vessels on her left thigh and was treated surgically.
